# Education interventions for health professionals on falls prevention in health care settings: a 10-year scoping review

**DOI:** 10.1186/s12877-020-01819-x

**Published:** 2020-11-09

**Authors:** L. Shaw, D. Kiegaldie, M. K. Farlie

**Affiliations:** 1 Faculty of Health Science, Youth and Community Studies, Holmesglen Institute, 488 South Road, Moorabbin, VIC 3189 Australia; 2 School of Allied Health, Human Services and Sport, La Trobe University, Bundoora, Victoria 3086 Australia; 3 Faculty of Health Science, Youth and Community Studies and Healthscope Hospitals, Holmesglen Institute, 488 South Road, Moorabbin, VIC 3189 Australia; 4 Eastern Clinical School, Faculty of Medicine, Nursing & Health Sciences, Monash University, Melbourne, Australia; 5 Department of Physiotherapy, School of Primary and Allied Health Care, Faculty of Medicine, Nursing and Health Sciences, Monash University, Moorooduc Highway, Frankston, VIC 3199 Australia

**Keywords:** Falls, Hospital, Health professional education, Prevention

## Abstract

**Background:**

Falls in hospitals are a major risk to patient safety. Health professional education has the potential to be an important aspect of falls prevention interventions. This scoping review was designed to investigate the extent of falls prevention education interventions available for health professionals, and to determine the quality of reporting.

**Method:**

A five stage scoping review process was followed based on Arksey and O’Malley’s framework and refined by the Joanna Briggs Institute Methodology for JBI Scoping Reviews. Five online databases identified papers published from January 2008 until May 2019. Papers were independently screened by two reviewers, and data extracted and analysed using a quality reporting framework.

**Results:**

Thirty-nine publications were included. Interventions included formal methods of educational delivery (for example, didactic lectures, video presentations), interactive learning activities, experiential learning, supported learning such as coaching, and written learning material. Few studies employed comprehensive education design principles. None used a reporting framework to plan, evaluate, and document the outcomes of educational interventions.

**Conclusions:**

Although health professional education is recognised as important for falls prevention, no uniform education design principles have been utilised in research published to date, despite commonly reported program objectives. Standardised reporting of education programs has the potential to improve the quality of clinical practice and allow studies to be compared and evaluated for effectiveness across healthcare settings.

## Background

Falls are one of the most serious safety problems in healthcare facilities worldwide, and are associated with marked morbidity, mortality, increased length of stay and re-admissions [1–5]. Falls can also incur substantial costs to hospitals and healthcare providers, insurers and individuals [6–11]. Despite extensive research on interventions designed to reduce the incidence of falls in hospitals, the quality of evidence is comparatively low, and the effects on falls risk in hospitals remains unclear [12, 13].

Education has been employed as a single intervention or as part of a multifactorial intervention in many falls prevention programs [12]. Much of the literature in this area has focussed on patient education in hospitals [3, 13–15], or elderly adults residing in the community or residential aged care [16–18]. Educating healthcare professionals about how to prevent falls has been recognised as a priority to improve patient safety in hospitals and residential care [9, 10]. There remains a need for targeted examination of the impact of education to health professionals in prevention of falls, using behavioural change models or theoretical frameworks and principles of good education design [19–21].

A recent Cochrane review [12] on interventions for preventing falls in older people in residential aged care and hospitals, evaluated three studies that reported the outcomes of staff training programs. It limited the assessment to reduction in falls rates, and did not report educational methods or educational outcomes [12]. For health professionals to develop the necessary knowledge, skills and attitudes required to deliver evidence-based care in the prevention of falls, there is a need to understand the best ways to structure and deliver staff falls education [22]. The details reported in studies of health professional education trials is therefore important, yet the quality of reporting has been inconsistent and lacked detailed description [23–27].

For clinical research trials, a number of reporting guidelines have been developed to support the completeness of reporting [24]. These include the Consolidated Standards of Reporting Trials (CONSORT) for randomised trials [28–31], and the Preferred Reporting Items for Systematic Review and Meta-Analyses statement (PRISMA) [32–34]. The PRISMA checklist was further developed for the reporting of scoping reviews, (PRISMA-ScR), to evaluate key items to be reported in scoping reviews. However, few education studies report whether conceptual frameworks guided development and implementation [20, 35]. Previous systematic reviews investigating the quality of reporting in medical and health professions education have found informative educational elements are sometimes missing, such as context, educational design, reporting of education outcomes, and reporting of limitations [23, 25–27]. Inadequate reporting of the key elements of education interventions could compromise the ability to replicate and apply the findings [24]. Falls prevention education programs for clinicians that do not employ a theoretical framework in the design process, administration protocols, and procedures of the intervention, might lack scientific rigour [20, 21]. This could compromise the effectiveness of the intervention and its application in clinical practice [20].

Complete reporting of education design can benefit from the employment of a learning model such as Biggs’ 3P model [36], which offers insights into the nature of learning. It describes teaching context, student approaches to learning and the outcomes of learning as a system [36]. Biggs’ integrated system comprises three components: Presage, Process and Product. Presage factors occur prior to learning and relate to the student (clinicians in this case) and teaching context [36]. Process factors are the processes that learners use to achieve tasks [36]. The Product phase is related to learning outcomes, with deep learning approaches expected to produce higher quality learning outcomes [36]. Kiegaldie (2015) suggested an extension to Biggs’ model, known as the 4Ps approach to education design, with the additional ‘P’ for Planning [35]. The inclusion of Planning emphasises the essential requirement for careful preparation and planning of education interventions. Presage and Planning go ‘hand in hand’, with Presage used to identify the issues/items, and Planning seen as the action plan to define what is needed to make the Presage happen [35]. The 4Ps approach is an iterative process, though equal attention is needed on every component [35]. Kiegaldie and Farlie (2019) proposed a quality tool for the design of education interventions [37] based on the extended 4Ps model. The conceptualisation of the 4Ps model as a checklist can assist evaluation of both education program quality and completeness of intervention reporting [37].

Given the limited reporting of a standard approach to health professional education on falls prevention, a scoping review was conducted to determine the nature of reported education programs. This scoping review aims to (i) investigate the extent of reporting of falls prevention education interventions for health professionals in a healthcare setting, (ii) appraise the quality of reporting of falls prevention education interventions using the 4Ps model of education design.

## Methods

We utilised the Arksey and O’Malley methodological framework [38] for scoping reviews, which was refined by the Joanna Briggs Institute [39]. The protocol was drafted using the PRISMA-ScR checklist [40], which was revised by the research team (LS, MF, DK). This checklist has five sections: (a) identifying the research question, (b) identifying relevant studies, (c) identifying the study selection criteria, (d) charting the data and (e) reporting the results. The first four stages are methodological and will be reported in this section, whereas the fifth stage will be reported in the results section of this review.

- Identifying the research question

The initial research question developed was, (i) What is the extent of education interventions delivered to health professionals (all those involved in caring for the individual including medical practitioners, nurses, allied health professionals and care facility staff), as a single intervention or as part of a multi-faceted intervention, that have been reported in the falls prevention literature? A secondary question was added to further focus the review, (ii) What is the quality of reporting of education interventions delivered to health professionals in the falls prevention literature? The authorship team consisted of researchers with clinical and educational expertise.

b) Identifying relevant studies

### Eligibility criteria

The population of interest was health professionals who had received education related to falls prevention. The concept of interest was staff education on falls prevention, and the context of interest was any hospital or healthcare setting. Healthcare settings were defined as acute or sub-acute hospitals, residential aged care facilities, rehabilitation facilities, or long-term care facilities. Falls prevention education interventions to health professionals in the community were excluded. To be included, articles needed to be peer-reviewed and in the English language. Included articles needed to describe primary research of any design (quantitative, qualitative and mixed methods), such as a cluster randomised controlled trial, quality improvement project, prospective cohort studies, pre-post and repeated measure designs, and quasi experimental studies. They needed to investigate falls prevention interventions including a health professions education component, as either a single or part of a multifactorial set of interventions. Our intent was to review interventions from countries with similar pedagogical approaches (i.e. Australia, New Zealand, Canada, the United States of America, or the United Kingdom), with student-centred classes and active participation in the learning and teaching process [41]. The articles had to be accessible as full text, and published between January 2008 and May 2019. Exclusions were websites, handouts or other types of passive educational materials, book chapters and literature reviews.

#### Search strategy

A three-step search strategy was developed by the study group in collaboration with an academic librarian. The librarian executed the searches on behalf of the study group:

(i) Initial search of PubMed and Cumulative Index to Nursing and Allied Health Literature (CINAHL), to identify relevant studies to assist with search term development, based on the research questions and purpose of the study. The librarian helped guide a rigorous analysis process to identify the best search terms and strategy related to education of health professionals on falls prevention in institutional settings. The process was iterative, to ensure all relevant search terms were captured.
(ii) Analysis of words in the title and abstract of the initial retrieved papers and indexing terms used to classify the articles.
(iii) Comprehensive search across PubMed, CINAHL, CENTRAL, PsycINFO and ERIC from January 2008 to May 2019, to ensure programs that were contemporary in terms of education design and falls prevention content. The reference lists of all identified studies were searched for additional studies meeting the inclusion criteria. We retrieved all supplementary files that were referred to in the included papers and any papers that were referred to in a particular study that were part of the research project.

Additional file 1 shows the complete search strategy executed in PubMed.

c) Study selection criteria

All studies identified from the search strategy were uploaded to Covidence [42]. Two reviewers (LS, MF) independently screened all titles and abstracts of retrieved papers. The same reviewers independently screened full texts to identify studies meeting the review criteria. Conflicts at each stage were resolved by discussion to consensus. If a consensus could not be reached, the third study group member (DK) was consulted. In all cases consensus was reached.

d) Charting the data

Data from eligible studies were charted independently by two researchers using a data extraction spreadsheet based on the 4Ps education design model (see Additional file 2) [37], which was developed as part of the study protocol. The tool captured the relevant information on key study characteristics, as well as Presage, Planning, Process and Product. The data extraction form was trialled by two reviewers (LS, MF) on three studies in duplicate to ensure that all relevant results were able to be captured. After which the same two reviewers independently charted the data for all included studies, and then compared and merged the data into a final dataset. Conflicts at the data merging stage were resolved by discussion to consensus. If a consensus could not be reached, the third study group member (DK) was consulted. In all instances consensus was reached.

## Results

**Fig. 1:**
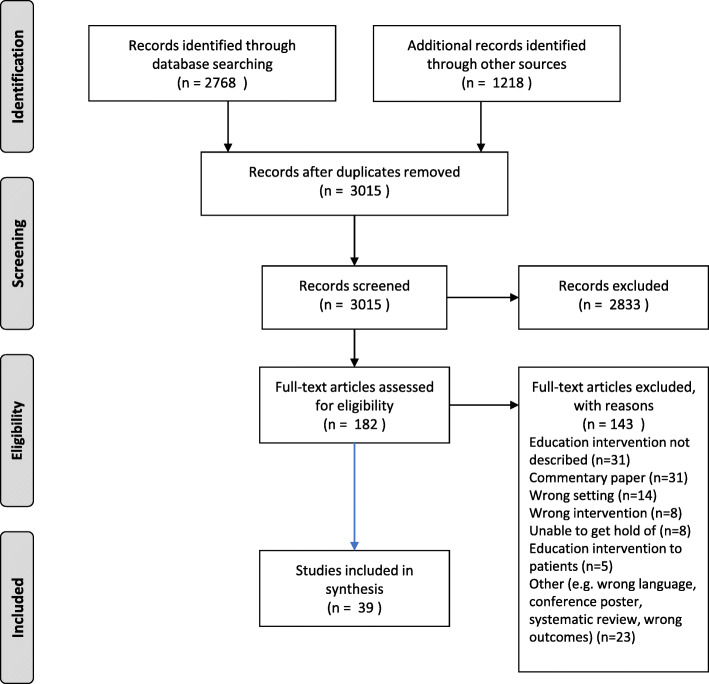
Prisma Diagram of Scoping Review results for education to health professionals

A summary of the key features of included studies are presented in Additional files 3, 4, 5, 6, 7. A total of 3015 records were retrieved from the 5 databases, following removal of duplicates. The results of the search strategy were charted using a PRISMA flow diagram (Fig. 1). On review of titles and abstracts 2833 records were identified as not meeting the inclusion criteria. Of those remaining, 182 full text articles were read and 143 were excluded. The most common reasons for exclusion were education intervention not described (n = 39), no education intervention reported (n = 31), commentary papers (n = 14), and wrong study setting (not healthcare or hospital) (n = 14). In summary, 39 articles were retained for this review.

### Study characteristics

Additional file 3 outlines the study characteristics including the authors, year published, study design and country in which the study was conducted. The majority of the studies were from the USA (n = 24), followed by Australia (n = 8), Canada (n = 3), Germany (n = 2), and the United Kingdom (n = 2).

### Types of study

Where study design was explicitly reported, seven reported the design as a randomised controlled trial [43–48]. Ten studies reported their design to be a pre-post study [49–58], one of these was reported as quasi-experimental [56], and six were cohort studies [51–55, 58]. One study was described as a quasi-experimental, pre-test/ post-test, pilot cohort [59]. Ten papers reported that their project was a quality improvement or implementation project, often advising that it was evidence based [46, 60–68]. Other study design descriptions included a multi-strategy interdisciplinary program implementation [69], education intervention [70], translational research intervention [71], or team training in a simulation environment [72].

### Presage and planning elements

These elements are outlined in Additional file 4, which include the learning environment, methods of recruitment for the teachers, details of teachers’ experience in education and falls prevention, and whether an evaluation was planned.

### Rationale for the use of education as an intervention

For the majority of studies, the rationale for conducting education programs was related to the high rates of falls in hospitals and residential aged care, particularly for those over 65 years of age. Many studies described the consequences associated with falls, including high morbidity and mortality, and associated high economic costs. Researchers noted a lack of healthcare professional knowledge, awareness and skills for implementing fall prevention strategies. Studies reported a need for educationally sound and evidence-based programs that engaged multiple professions in interactive learning and clinically relevant problem solving to achieve high quality patient-centred care.

### Purpose of education interventions

The purpose of education interventions was primarily described as increasing health professionals’ knowledge, attitudes, skills and behaviour related to falls prevention, and determine whether health professions training had an effect on falls and injury rates. Some studies also reported aiming to improve interprofessional collaboration, communication and teamwork in managing falls. Most studies highlighted the importance of promoting adherence to current best practice falls prevention strategies. They also noted the value of ensuring that the safety education curriculum developed was evidence based.

### Study location

Over two-thirds (n = 23) of the studies were conducted in a hospital. Nine were in residential aged care facilities, and three studies were conducted in a combined setting. The majority of studies were conducted ‘in house’ though the actual learning environment was not stated. A small number were conducted on wards, in classrooms, or in simulation centres.

### Resources

Table 1 outlines the reported resources used to deliver the education. Most studies (n = 36) outlined the resources required to deliver the education program. In studies where this was not explicitly described, ‘didactic learning materials’ or ‘practical workshop resources’ were reported [44, 45, 69]. Some identified various support resources, brochures, or handouts, summarising the session and key learning points. One study described supplying a pack incorporating information from international best practice guidelines [58]. Another sent a support package to participants before the education intervention that included a copy of the presentation slides, reference to further readings, and a DVD of the assessment procedures to be covered [47]. One study provided a fall bundle toolkit that included a patient communication board, patient and family teaching materials, and related forms [78]. Of those studies that employed video, one video was a demonstration of an intervention [71], another study used video conferencing facilities to deliver falls prevention education to clinicians [75].

**Table 1:** Resources used to deliver the education

Resources	No. of papers	Paper ID
Didactic learning materials/ workshop resources	3	[44, 45, 69]
Support resources, brochures, or handouts	10	[44–46, 48, 53, 67, 70, 71, 73, 74]
Resources required to deliver in-house education/ team presentations	3	[46, 69, 71]
Videos (support written material/ case studies/ clinical scenarios)	9	[47, 51, 63, 71, 73–77]
Presentation slides and visual aids	13	[43, 46–49, 57, 59, 64, 66, 77–80]
Online education modules	6	[44, 45, 62, 68, 74, 80]
Role playing or case studies	9	[44, 45, 51, 59, 67, 69, 73, 80, 81]
Simulation	2	[60, 72]
Knowledge surveys	4	[52–54, 72]
Evaluation and feedback surveys	2	[57, 81]

A range of facilities across the studies were used to deliver the training. These included seminar rooms, tutorial rooms, and training centres.

### Who taught the education program?

The education programs were taught by a variety of educators, although it was not always clear who delivered the intervention. Around one-quarter of studies utilised nursing staff, who often had some expertise in falls prevention [46, 49, 56, 58–60, 70, 71, 77, 78, 80]. Other studies employed an interprofessional team, who were usually nominated based on their knowledge of falls prevention, commitment to patient safety or clinical skills [53, 55, 61, 63, 65, 75, 79, 81, 82]. One study reported employing a local expert in the field who had previously published in the area of falls prevention [47]. Four studies reported using trained interventionists to deliver the education, including change agents and falls ‘champions’ [44, 45, 71, 79]. Research team members (including project representatives) were the educators in around one-quarter [43, 46, 48, 51, 52, 54, 64, 68, 69, 76]. Geriatrician clinical educators were the educators in two studies [67, 73], and where the study was carried out in a simulation centre, the simulation centre staff were the educators [60, 72]. Two studies involved self-directed learning [50, 74] and one study did not state who the educator was [62].

### Who were the learners?

Uni-professional education programs were usually delivered to nursing staff, though three studies delivered education to medical staff or medical students [52, 69, 83]. One study reported interprofessional education to nursing and allied health staff, while junior doctors attended a separate session with greater emphasis on diagnosis and treatment of underlying conditions, run by a senior geriatrician [59]. Some studies stated that education was to all employees, or care facility staff but did not state whether they were clinical or non-clinical. Many studies reported educating all clinical staff involved in the care of the patient, including (but not limited to) nurses, physicians, social workers, physiotherapists, occupational therapists, speech therapists, pharmacists, dietitians, and healthcare aides. Five studies reported inclusion of non-professional clinical and support staff in their program delivery, including, for example, environmental services, maintenance, housekeepers, clerical staff, students, porters and laboratory and diagnostic technicians [44–46, 53, 74].

### How many learners were educated?

Table 2 states how many learners were included in the education intervention. Eleven studies did not state how many learners were educated or it was unclear [48, 58, 61, 62, 66, 67, 69, 71, 74, 75, 78]. One study only reported the percentage of staff trained [49]. Another reported educating ‘change agents’ from 256 nursing homes but not the final number educated [71]. For some studies, there appeared to be a gradual attrition rate from the start of the study, to the completion of the educational content and subsequent completion of post study surveys.

**Table 2:** Number of learners in the education intervention

Number of learners	No. of papers	Paper ID
0–10	2	[51, 57]
10–50	7	[52, 54, 60, 70, 72, 77, 81]
51–100	11	[50, 53, 55, 56, 59, 63–65, 68, 79, 83]
100–200	3	[46, 47, 80]
> 200 (300, 471, 658)	3	[44, 45, 76]

### Process elements

Additional file 5 describes the Process elements of educational interventions. Twenty-one studies reported that teachers were trained in how to deliver the program. Twenty-six studies reported that there were pre-determined learning objectives. Of these, fourteen studies reported their objectives in behavioural terms. Eleven studies explicitly reported recognising learners’ prior knowledge and a further eight studies appeared to informally recognise prior knowledge. Twenty-five studies reported some recognition of learners’ prior experience. Three studies did not state the learning and teaching methods employed and three studies had no apparent alignment between the learning and teaching methods and their learning objectives.

### Teaching and learning process

A range of teaching and learning activities were conducted across the studies and these are detailed in Additional file 6. The approaches employed for educating staff about falls prevention mainly focused on three larger categories: methods of delivery, interactive learning activities/ experiential learning, and supported learning. There were three other minor categories: written learning material, assessments and ‘other’ which included teleconferences. These categories are detailed in Table 3. Often studies engaged a number of approaches.

**Table 3:** Categories of teaching and learning approaches

Teaching/ learning category	Sub categories
*Methods of delivery*	Didactic lectures/ formal delivery
	Other oral presentation e.g. in-service training
	E-learning/ online
	Self-directed learning
	Video presentation/ demonstration
*Interactive learning activities/ experiential learning*	Group -based learning activities (e.g. team presentations, problem solving, brainstorming)
	Debriefing sessions/ reflective dialogue
	Station-based activities
	Case studies/ clinical scenarios (paper-based)
	Case studies/ clinical scenarios (video)
	Role play
	Simulation
	Skills training
	Interactive learning activities (details not described)
*Supported learning*	Individual mentoring/ coaching or personal feedback
	Bedside coaching
	Peer to peer discussion and feedback/ staff huddles
	Staff meetings
	Team coaching
*Written learning material*	Handouts
	Resource folders
	Falls assessment tool
	Poster
*Assessments*	Practical assessment
	Knowledge assessment
*Other*	Teleconferences

#### Methods of delivery

Didactic lectures and formal delivery of content was the most commonly reported method of education to health professionals and was employed as a method of teaching in over half (n = 22) of the studies. Other methods of delivery utilised included in-service training (n = 8), online teaching (n = 8), self-directed learning (n = 8) or video presentations and demonstrations (n = 8).

#### Interactive learning activities/ experiential learning

Many of the studies supported formal content delivery with interactive learning activities. Around half (n = 19) utilised group based learning activities, which included team presentations, problem solving and brainstorming. For example, in one study, participants rotated through four group learning stations in sixty minutes and completed a number of group tasks. These included identifying falls risk factors by synthesising data from a history and physical examination in a written clinical case study, and observing and documenting abnormal physical findings on gait videos [73]. Another commonly employed experiential learning method utilised by seventeen of the studies, was debriefing and reflective dialogue. The trial by Bursiek et al. (2017), presented an interdisciplinary simulation training scenario on patient falls, which was followed by a debriefing session and engagement of participants in reflective dialogue [72]. Participants in another study discussed the falls that had occurred on the patient care unit during the month at a falls meeting. This meeting included a discussion, brainstorming and reflection session about interventions that might work for the particular situations being discussed [56]. Fifteen of the studies included specific skills practice sessions, for example screening for falls, assessing gait, balance, orthostatic and other medical conditions, and often these sessions included opportunities for feedback.

#### Supported learning

Methods of supported learning reported in the studies included individual mentoring, bedside coaching, personal feedback or team coaching. Peer to peer discussion and feedback was reported as part of the teaching and learning process in over one-quarter (n = 11). One study reported multiple points of contact for peer-to-peer education such as at staff meetings, during start of shift huddles, via online education, and at ‘Practice Council’ meetings, to increase the likelihood of infusion of the proposed changes into real practice [62]. Another nine studies reported utilising individual mentoring, coaching or personal feedback. For example, participants in one study received 2 days of interactive team training followed by 3 months of coaching learners to implement their projects and share their stories and solutions with other teams [46].

#### Written learning material

The category of written learning material included teaching related to a falls assessment tool (n = 6). One study involved presenting information about the falls risk assessment tool to nurses, followed by discussion about how the tool and suggested interventions could be implemented at each of the sites [64]. Handouts and resource folders were utilised by some studies and one reinforced the falls prevention message via a poster for each session, which was displayed on a fall wall on each nursing unit [56].

#### Assessment of learning

One study reported assessing clinicians’ practical skills [69]. Six studies assessed participants’ knowledge, such as Haralambous and colleagues who tested knowledge of falls prevention risk factors and prevention interventions [58].

### Product elements

Additional file 7 describes the Product elements of educational design. Thirty-two studies evaluated clinical outcomes, and twenty-seven studies evaluated educational outcomes. Thirty studies assessed learners’ achievements of the learning objectives of stated purpose of the education program and twenty-nine studies conducted an evaluation of the education program. Data reported to evaluate the educational interventions included: pre and post knowledge tests; use of validated scales such as the Environment Assessment Scale, Mayo High Performance Teamwork Scale, Perceived Quality of Care Scale, and Safety Organizing Scale; ongoing process evaluation; observation of falls prevention interventions implemented post-education; questionnaires targeting knowledge change and practice change; and analysis of focus groups. Where clinical data was used to evaluate the education interventions, this was usually fall rates per 1000 bed days.

### Quality of health professions education programs

Using a checklist based on the expanded 4Ps model, a summary table of a number of quality metrics was created, including whether the resources required were outlined, teacher and learner characteristics and evaluation planning (Table 4).

**Table 4:** Quality scores for health professional education programs

Lead Author	Study location	Learning environment	Resources required outlined	^**a**^Teacher characteristics (/ 4)	^**b**^Learners characteristics (/ 3)	^**c**^Evaluation planned and executed? (/2)
Atkinson (2014) [73]	Other (AGS Conference)	Other -workshop at conference	✓	4	3	2
Becker (2011) [71]	LTC/RACF only	LCF	✓	2	2	–
Brennan (2018) [70]	Hospital only	Blended learning	✓	3	3	2
Bursiek (2017) [72]	Hospital only	Simulation centre	✓	1	3	2
Cabilan (2014) [49]	Hospital only	Ward or independent learning package	✓	2	3	1
Campbell (2016) [78]	Hospital only	Ward	✓	3	2	2
Caton (2011) [69]	Hospital only	In-house education program	✓	4	2	2
Colon-Emeric (2017) [44]	LTC/RACF only	Blended learning	✓	3	3	2
Colon-Emeric (2013) [45]	LTC/RACF only	Blended learning	✓	3	3	2
Dilley (2014) [76]	LTC/RACF and community	Other -in-house education program	✓	4	3	–
Eckstrom (2016) [79]	LTC/RACF and Hospital	Workshop plus coaching	✓	4	2	2
Godlock (2016) [60]	Hospital only	Simulation centre	✓	3	3	2
Gray-Miceli (2016) [46]	Hospital only	In-house education program	✓	3	3	–
Gygax Spicer (2017) [61]	Hospital only	Ward	✓	4	2	–
Haralambous (2010) [58]	LTC/RACF only	In-house education program	✓	2	2	1
Heck (2014) [62]	Hospital only	Blended learning	✓	4	2	–
Hill (2015) [75]	Hospital only	In-house education program	✓	2	1	–
Ireland (2010) [74]	Hospital only	Blended learning	✓	2	2	2
Johnson (2015) [50]	Hospital only	Online e-learning	✓	3	3	2
Karnes (2011) [51]	Outpatient rehabilitation in hospital	In-house education program	✓	2	3	2
Kempegowda (2018) [52]	Hospital only	Interprofessional workshop	✓	4	3	2
Kent (2018) [81]	Hospital only	Interprofessional workshop	✓	2	3	2
Lasater (2016) [63]	Other	Classroom	✓	4	3	2
Leverenz (2018) [57]	LTC/RACF only	In-house education program	✓	3	3	2
Lopez-Jeng (2019) [53]	Hospital only	In-house education program	✓	3	3	2
Lugo (2014) [54]	Hospital only	In-house education program	✓	2	3	2
Maloney (2011) [47]	Hospital, LTC/RACF and Community	Blended learning	✓	3	3	2
McCarty (2018) [64]	Hospital only	In-house education program	✓	2	3	1
McConnell (2009) [80]	Hospital only	Blended learning	✓	3	3	2
McKenzie (2017) [65]	LTC/ RACF and hospital	Classroom	-	2	3	2
Melin (2018) [66]	Hospital only	Face to face or online learning	✓	2	2	1
Meyer (2009) [48]	LTC/RACF only	In-house education program	✓	4	2	1
Singh (2016) [67]	Hospital only	In-house education program	✓	3	2	2
Spiva (2014) [77]	Hospital only	In-house education program	✓	4	3	2
Szymaniak (2015) [68]	Hospital only	Blended learning	✓	3	3	2
Teresi (2013) [43]	LTC/RACF only	In-house education program	✓	2	3	2
Toye (2017) [59]	Hospital only	In-house education program	✓	4	3	2
Wheeler (2018) [55]	LTC/RACF only	Other	-	4	3	2
Williams (2011) [56]	Hospital	Ward	✓	4	3	2

^a^**Teacher characteristics (4):** Who taught the education program? How were the teachers identified/recruited? Were the teachers qualified and/or experienced in teaching? Were the teachers qualified and/or experienced in the topic of falls prevention (subject matter experts)?

^b^**Learner characteristics (2):** Who were the learners? What was the configuration of the audience? How many learners were educated?

^c^**Evaluation planned (2):** Was there an assessment of the learners’ achievement of the learning objectives or stated purpose of the education program? Was an evaluation of the education program conducted?

## Discussion

This scoping review based on 39 studies published from January 2008 to May 2019, provides a comprehensive review of studies that have investigated education to health professionals on falls prevention in hospitals and healthcare settings. We identified a limited number of studies that primarily focussed on describing education interventions to health professionals on falls prevention, either as a single intervention or as part of a multifactorial organisational strategy. The overall finding was that the rigour of design and reporting of clinician educational interventions for falls prevention are often not comprehensive.

The evidence synthesis in this review was complex due to wide variation in the methods and quality of reporting, and extensive variability in educational approaches, rationale, purposes and methods of evaluation. Of the 182 full text articles that were screened to determine their suitability for this study, thirty-nine were rejected as the education intervention was not described. Previous reviews have likewise identified that education intervention reporting is inconsistent and often incomplete [23, 25–27, 84]. For example, studies evaluating education interventions related to cancer pain, found deficiencies in the extent and quality of reporting, with many studies lacking detailed descriptions of the format and content of their education programs [85, 86]. A review of simulation research for health professions education also noted that studies often failed to describe the context of the research, instructional design and outcomes [23]. In the reviewed studies, deficiencies in reporting were common with authors providing few details about the content of their education programs, which made it difficult to categorise and interpret the findings. Clear and concise reporting of education interventions helps readers understand how the education was delivered in the research [84]. Poor and inconsistent reporting of education interventions makes it difficult to interpret results and replicate interventions [84]. Hence it is less likely the research will inform change that will positively influence target outcomes [84].

### Presage and planning elements

None of the studies we evaluated used a quality framework to design their intervention, and few studies reported the different elements required for developing and reporting an education intervention. Inadequately describing the key elements of a research study means that others are unable to apply and replicate the methods [87]. For example, a core principle of education interventions is the educational dose intensity [88]. However, in the studies that we reviewed, the duration of the education interventions, the learning environment and other relevant information to characterise the dose was often difficult to find.

Use of the 4Ps framework [37], may assist a quality assurance process where all key elements are considered in the design and reporting of health professional education programs. This has been used successfully in other health professional contexts such as in interprofessional learning [89–91] and simulation-based education (SBE) [84]. In the SBE context, Cheng and colleagues argue for an improvement in the quality of reporting for SBE and have developed and published guidelines for healthcare simulation research inclusive of educational design features [84]. The use of standardised reporting of education design according to these types of frameworks will focus attention to the important elements for quality improvement into the future.

### Process elements –content of the education interventions

We found discrepancies in the content of education programs in studies with multiple teaching and learning strategies employed, which made the efficacy of each component difficult to determine [86]. Additionally, education interventions were poorly described, limiting the ability of the reader to fully understand the process, as well as making replication challenging. Formal delivery was the most common teaching strategy. Studies optimising health professions education in other diseases such as heart failure [92] and cancer [88] have demonstrated the importance of active learning for adult learners to improve their self-efficacy and level of knowledge of the disease. A scoping review that examined concussion education programs found that the education programs had limited use of interactive tools, delivered education at one time point only and lacked long-term assessment [93]. Our review revealed that whilst didactic lectures was the most common form of delivery, this was usually combined with other interactive learning activities, including skills training, or supported learning, with feedback or coaching. The time spent on education also varied greatly making it difficult to determine the most efficient and cost effective manner [86]. Many delivered education at one time point only, whilst others recognised the importance of follow up and reinforcement sessions via team meetings, teleconferences, peer to peer feedback or bedside coaching. More research is needed to determine the education program processes that could improve participants’ long term knowledge, attitudes and behaviours after being exposed to a falls education program [93].

### Product –outcomes and evaluation

Primarily, the outcomes were often measured in clinical terms, related to the number of falls, rather than behaviour change. The methods employed for outcome measurement also varied with quantitative instruments such as surveys, quizzes and questionnaires being the most common evaluation tools. The wide variety of approaches make it difficult to compare studies. Using robust and validated outcome measures will improve this field. Recommendations made on reporting outcome measures for cancer pain educational interventions, stated that all study designs should report on the prospectively selected primary outcome, and the tools and tests used to achieve this [88].

Evaluation of clinician training is often considered to be a low priority [94]. Application of the extended 4Ps model [37] to the studies in this review of education interventions in falls prevention, has provided stratified assessment of the use of education evaluation which highlights stronger study designs without unnecessarily discounting partially helpful information [95]. Evaluating the behavioural outcomes of education programs is important given that behaviour change is an important goal of the education. It is therefore recommended that the primary endpoints for research on health professional education programs in falls prevention should not only focus on falls and injury rates and costs. The clinical assumption of patient benefit as a reference standard of evidence should be rejected [95] and we call for researchers to also measure behavioural outcomes. Effective training measured in terms of behavioural change, such as the transfer of knowledge and skills gained from training into practice [88, 96], may potentially lead to a reduction in the rate of falls. The evaluation of education interventions using qualitative and quantitative measures could be incorporated into future falls prevention education programs for health professionals [95].

### Limitations

Including the synthesis of qualitative and quantitative research in the same review [97], and balancing the breadth and depth of analysis [98], was challenging. The sources of evidence for this review are limited because we excluded articles that were not published in countries with similar pedagogical approaches, only reported on falls prevention to health professionals in hospitals or healthcare facilities, and excluded non-empirical studies. Reporting of training undertaken for the teachers or facilitators of the education interventions could be considered as an addition to the framework in future studies. The application of the extended 4Ps model as a quality assessment tool for evaluation of educational reporting was theoretically driven. The 4Ps model awaits further formal validation [37].

## Conclusions

Our scoping review highlighted gaps in the planning, reporting and evaluation processes for health professional education in falls prevention. It also generated a recommendation to adopt a more comprehensive approach. We found a variety of methods for education of health professionals in falls prevention. Investigation and reporting of well-designed education programs for health professionals on falls prevention in institutional settings is needed to determine the effectiveness of this type of intervention for falls prevention. Use of a standardised reporting framework for education interventions in falls prevention research, such as the extended 4Ps model, has the potential to improve knowledge and prevent falls.

## Supplementary information

**Additional file 1.** Example search strategy PUBMED

**Additional file 2.** Modified 4Ps model of quality in education design

**Additional file 3.** Study characteristics

**Additional file 4.** Presage and planning elements of education interventions

**Additional file 5.** Process elements of education interventions

**Additional file 6.** Teaching and learning activities

**Additional file 7.** Product elements of education interventions

## Data Availability

The datasets used and/or analysed during the current study are available from the corresponding author on reasonable request.

## Abbreviations

JBI: Joanna Briggs Institute Methodology
CONSORT: Consolidated Standards of Reporting Trials
PRISMA: Preferred Reporting Items for Systematic Review and Meta-Analyses statement
PRISMA-ScR: Preferred Reporting Items for Systematic Review and Meta-Analyses statement for scoping reviews
Biggs’ 3P model: Presage, Process and Product
Kiegaldie’s 4Ps model: Presage, Process, Product and Planning
LS: Louise Shaw
MF: Melanie Farlie
DK: Debra Kiegaldie
CINAHL: Cumulative Index to Nursing and Allied Health Literature
CENTRAL: The Cochrane Central Register of Controlled Trials
ERIC: Education Resources Information Center
SBE: Simulation Based Education
