# Supplementary material for: Education interventions for health professionals on falls prevention in health care settings: a 10-year scoping review
Source: BMC Geriatr. 2020 Nov 9;20:460. doi: 10.1186/s12877-020-01819-x (PMC7653707; doi:10.1186/s12877-020-01819-x)
Supplement: Supplementary file 1 — Additional file 1. Example search strategy PUBMED [file 12877_2020_1819_MOESM1_ESM.docx]

**Additional file 1: Example search strategy PUBMED**

| S1 | Accidental falls[MESH] | 22108 |
| --- | --- | --- |
| S2 | "curriculum"[MeSH Terms] OR "education, professional"[MeSH Terms] OR "inservice training"[MeSH Terms] OR "education"[Subheading] OR “teaching”[Mesh] | 472260 |
| S3 | S1 AND S2 | [436](https://www.ncbi.nlm.nih.gov/pubmed/?term=(((%22curriculum%22%5BMeSH+Terms%5D+OR+%22education%2C+professional%22%5BMeSH+Terms%5D+OR+%22inservice+training%22%5BMeSH+Terms%5D+OR+%22education%22%5BSubheading%5D+OR+%E2%80%9Cteaching%E2%80%9D%5BMesh%5D)))+AND+accidental+falls%5BMesh%5D) |
| S4 | Fall[TI] OR Falls[TI] OR Falling[TI] OR faller*[TI] | 19178 |
| S5 | prevent*[TIAB] OR risk*[TIAB] OR reduc*[TIAB] OR improv*[TIAB] OR decreas*[TIAB] OR rate[TIAB] | 9051399 |
| S6 | S4 AND S5  (Fall[TI] OR Falls[TI] OR Falling[TI] OR faller*[TI]) AND (prevent*[TIAB] OR risk*[TIAB] OR reduc*[TIAB] OR improv*[TIAB] OR decreas*[TIAB] OR rate[TIAB]) | 11246 |
| S7 | personnel [TIAB] OR nurse [TIAB] OR nurses[TIAB] OR staff [TIAB] OR worker*[TIAB] OR workforce[TIAB] OR "allied Health"[TIAB] OR physiotherap*[TIAB] OR occupational [TIAB] OR assistant*[TIAB] OR team*[TIAB] OR teamwork[TIAB] OR professional* [TIAB] OR practitioner*[TIAB] OR multi[TIAB] OR multifac*[TIAB] OR multicomp*[TIAB] OR multidis*[TIAB] OR multimodal[TIAB] OR interprofessional[TIAB] OR interdisciplinary[TIAB] | 1483758 |
| S8 | educat*[TIAB] OR knowledge[TIAB] OR instruct*[TIAB] OR skill*[TIAB] OR session*[TIAB] OR "evidence based"[TIAB] OR train*[TIAB] OR information [TIAB] | 2702072 |
| S9 | S6 AND S7 AND S8  (Fall[TI] OR Falls[TI] OR Falling[TI] OR faller*[TI]) AND (prevent*[TIAB] OR risk*[TIAB] OR reduc*[TIAB] OR improv*[TIAB] OR decreas*[TIAB] OR rate[TIAB]) AND (personnel [TIAB] OR nurse [TIAB] OR nurses[TIAB] OR staff [TIAB] OR worker*[TIAB] OR workforce[TIAB] OR "allied Health"[TIAB] OR physiotherap*[TIAB] OR occupational [TIAB] OR assistant*[TIAB] OR team*[TIAB] OR teamwork[TIAB] OR professional* [TIAB] OR practitioner*[TIAB] OR multi[TIAB] OR multifac*[TIAB] OR multicomp*[TIAB] OR multidis*[TIAB] OR multimodal[TIAB] OR interprofessional[TIAB] OR interdisciplinary[TIAB]) AND (educat*[TIAB] OR knowledge[TIAB] OR instruct*[TIAB] OR skill*[TIAB] OR session*[TIAB] OR "evidence based"[TIAB] OR train*[TIAB] OR information [TIAB])) | [1147](https://www.ncbi.nlm.nih.gov/pubmed/?term=(Fall%5BTI%5D+OR+Falls%5BTI%5D+OR+Falling%5BTI%5D+OR+faller*%5BTI%5D)+AND+(prevent*%5BTIAB%5D+OR+risk*%5BTIAB%5D+OR+reduc*%5BTIAB%5D+OR+improv*%5BTIAB%5D+OR+decreas*%5BTIAB%5D+OR+rate%5BTIAB%5D)+AND+(personnel+%5BTIAB%5D+OR+nurse+%5BTIAB%5D+OR+nurses%5BTIAB%5D+OR+staff+%5BTIAB%5D+OR+worker*%5BTIAB%5D+OR+workforce%5BTIAB%5D+OR+%22allied+Health%22%5BTIAB%5D+OR+physiotherap*%5BTIAB%5D+OR+occupational+%5BTIAB%5D+OR+assistant*%5BTIAB%5D+OR+team*%5BTIAB%5D+OR+teamwork%5BTIAB%5D+OR+professional*+%5BTIAB%5D+OR+practitioner*%5BTIAB%5D+OR+multi%5BTIAB%5D+OR+multifac*%5BTIAB%5D+OR+multicomp*%5BTIAB%5D+OR+multidis*%5BTIAB%5D+OR+multimodal%5BTIAB%5D+OR+interprofessional%5BTIAB%5D+OR+interdisciplinary%5BTIAB%5D)+AND+(educat*%5BTIAB%5D+OR+knowledge%5BTIAB%5D+OR+instruct*%5BTIAB%5D+OR+skill*%5BTIAB%5D+OR+session*%5BTIAB%5D+OR+%22evidence+based%22%5BTIAB%5D+OR+train*%5BTIAB%5D+OR+information+%5BTIAB%5D)) |
| S10 | S3 OR S9 | [1481](https://www.ncbi.nlm.nih.gov/pubmed/?term=(((((%22curriculum%22%5BMeSH+Terms%5D+OR+%22education%2C+professional%22%5BMeSH+Terms%5D+OR+%22inservice+training%22%5BMeSH+Terms%5D+OR+%22education%22%5BSubheading%5D+OR+%E2%80%9Cteaching%E2%80%9D%5BMesh%5D)))+AND+accidental+falls%5BMesh%5D))+OR+(((Fall%5BTI%5D+OR+Falls%5BTI%5D+OR+Falling%5BTI%5D+OR+faller*%5BTI%5D)+AND+(prevent*%5BTIAB%5D+OR+risk*%5BTIAB%5D+OR+reduc*%5BTIAB%5D+OR+improv*%5BTIAB%5D+OR+decreas*%5BTIAB%5D+OR+rate%5BTIAB%5D)+AND+(personnel+%5BTIAB%5D+OR+nurse+%5BTIAB%5D+OR+nurses%5BTIAB%5D+OR+staff+%5BTIAB%5D+OR+worker*%5BTIAB%5D+OR+workforce%5BTIAB%5D+OR+%22allied+Health%22%5BTIAB%5D+OR+physiotherap*%5BTIAB%5D+OR+occupational+%5BTIAB%5D+OR+assistant*%5BTIAB%5D+OR+team*%5BTIAB%5D+OR+teamwork%5BTIAB%5D+OR+professional*+%5BTIAB%5D+OR+practitioner*%5BTIAB%5D+OR+multi%5BTIAB%5D+OR+multifac*%5BTIAB%5D+OR+multicomp*%5BTIAB%5D+OR+multidis*%5BTIAB%5D+OR+multimodal%5BTIAB%5D+OR+interprofessional%5BTIAB%5D+OR+interdisciplinary%5BTIAB%5D)+AND+(educat*%5BTIAB%5D+OR+knowledge%5BTIAB%5D+OR+instruct*%5BTIAB%5D+OR+skill*%5BTIAB%5D+OR+session*%5BTIAB%5D+OR+%22evidence+based%22%5BTIAB%5D+OR+train*%5BTIAB%5D+OR+information+%5BTIAB%5D)))) |
| S11 | S10 limited to 2008 onwards | [1086](https://www.ncbi.nlm.nih.gov/pubmed/?term=(((((%22curriculum%22%5BMeSH+Terms%5D+OR+%22education%2C+professional%22%5BMeSH+Terms%5D+OR+%22inservice+training%22%5BMeSH+Terms%5D+OR+%22education%22%5BSubheading%5D+OR+%E2%80%9Cteaching%E2%80%9D%5BMesh%5D)))+AND+accidental+falls%5BMesh%5D))+OR+(((Fall%5BTI%5D+OR+Falls%5BTI%5D+OR+Falling%5BTI%5D+OR+faller*%5BTI%5D)+AND+(prevent*%5BTIAB%5D+OR+risk*%5BTIAB%5D+OR+reduc*%5BTIAB%5D+OR+improv*%5BTIAB%5D+OR+decreas*%5BTIAB%5D+OR+rate%5BTIAB%5D)+AND+(personnel+%5BTIAB%5D+OR+nurse+%5BTIAB%5D+OR+nurses%5BTIAB%5D+OR+staff+%5BTIAB%5D+OR+worker*%5BTIAB%5D+OR+workforce%5BTIAB%5D+OR+%22allied+Health%22%5BTIAB%5D+OR+physiotherap*%5BTIAB%5D+OR+occupational+%5BTIAB%5D+OR+assistant*%5BTIAB%5D+OR+team*%5BTIAB%5D+OR+teamwork%5BTIAB%5D+OR+professional*+%5BTIAB%5D+OR+practitioner*%5BTIAB%5D+OR+multi%5BTIAB%5D+OR+multifac*%5BTIAB%5D+OR+multicomp*%5BTIAB%5D+OR+multidis*%5BTIAB%5D+OR+multimodal%5BTIAB%5D+OR+interprofessional%5BTIAB%5D+OR+interdisciplinary%5BTIAB%5D)+AND+(educat*%5BTIAB%5D+OR+knowledge%5BTIAB%5D+OR+instruct*%5BTIAB%5D+OR+skill*%5BTIAB%5D+OR+session*%5BTIAB%5D+OR+%22evidence+based%22%5BTIAB%5D+OR+train*%5BTIAB%5D+OR+information+%5BTIAB%5D)))) |
