# Supplementary material for: Education interventions for health professionals on falls prevention in health care settings: a 10-year scoping review
Source: BMC Geriatr. 2020 Nov 9;20:460. doi: 10.1186/s12877-020-01819-x (PMC7653707; doi:10.1186/s12877-020-01819-x)
Supplement: Supplementary file 2 — Additional file 2. Modified 4Ps model of quality in education design [file 12877_2020_1819_MOESM2_ESM.docx]

**Additional file 2: Modified 4Ps model of quality in education design**

| **Item** | **Key questions** |
| --- | --- |
| **Pressage/ Planning** | What was the rationale for the education program? |
|  | What was the purpose of the education program? |
|  | What was the learning environment? |
|  | What resources were required to deliver the program? |
|  | Who taught the education program? |
|  | How were the teachers identified/recruited? |
|  | Were the teachers qualified and/or experienced in teaching? |
|  | Were the teachers qualified and/or experienced in the topic of falls prevention (subject matter experts)? |
|  | Who were the learners? |
|  | What was the configuration of the audience? |
|  | How many learners were educated? |
|  | Was an evaluation of the education program planned from the inception of the development of the program? |
| **Process** | Were the teachers trained in how to deliver the education program? |
|  | Were the learning objectives for the education program stated? |
|  | Were learning objectives written in behavioural terms? |
|  | Was there recognition of learners' prior knowledge? |
|  | Was there recognition of learners' prior experience? |
|  | Were the teaching & learning methods stated? |
|  | What did the learners do in the education program? |
|  | What did the teachers do in the education program? |
|  | Were the teaching & learning methods constructively aligned to the learning objectives or the stated purpose of the education program? |
|  | How long did the education program take to complete? |
| **Product** | What types of outcomes evaluated? |
|  | Was there an assessment of the learners' achievement of the learning objectives or stated purpose of the education program? |
|  | Was an evaluation of the education program conducted? |
|  | How was the evaluation data gathered? |
|  | What level/s of evaluation are reported? |
