# Supplementary material for: Education interventions for health professionals on falls prevention in health care settings: a 10-year scoping review
Source: BMC Geriatr. 2020 Nov 9;20:460. doi: 10.1186/s12877-020-01819-x (PMC7653707; doi:10.1186/s12877-020-01819-x)
Supplement: Supplementary file 3 — Additional file 3. Study characteristics [file 12877_2020_1819_MOESM3_ESM.docx]

**Additional file 3: Study characteristics**

| **Authors** | **Year** | **Title** | **Study Design** | **Country** |
| --- | --- | --- | --- | --- |
| Atkinson, H.H.; Tan, ZS.; Brennan, M.; et al | 2014 | A Collaborative National Model to Assess Competencies for Medical Students, Residents, and Other Healthcare Practitioners in Gait and Falls Risk Evaluation | Curriculum/ teaching model | USA |
| Becker, C.; Cameron, I. D.; Klenk, J.; et al | 2011 | Reduction of femoral fractures in long-term care facilities: the Bavarian fracture prevention study | Translational intervention study based on previous RCT | Germany |
| Brennan, D. | 2018 | Patient Self-Assessment for Older Adult Fall Prevention | Educational intervention study (Doctor of Nursing Practice thesis) | USA |
| Bursiek, A. A.; Hopkins, M. R.; Breitkopf, D. M.; et al | 2017 | Use of High-Fidelity Simulation to Enhance Interdisciplinary Collaboration and Reduce Patient Falls | Pilot study of team training in simulation environment | USA |
| Cabilan, C.J. | 2014 | Falls risk assessment and falls prevention strategies in private oncology and neurosurgical setting: a best practice implementation project | Evidence-based pre-post implementation audit project. JBI implementation report | Australia |
| Campbell, B | 2016 | Fall Safety Bundle | Development of a falls safety bundle and falls prevention curriculum | USA |
| Caton, C.; Wiley, M. K.; Zhao, Y.; et al | 2011 | Improving internal medicine residents' falls assessment and evaluation: an interdisciplinary, multistrategy program | Multi-strategy interdisciplinary program implementation | USA |
| Colon-Emeric, C..S.; Corazzini, K.; McConnell, E.S.; et al | 2017 | Effect of Promoting High-Quality Staff Interactions on Fall Prevention in Nursing Homes: A Cluster-Randomized Trial | Cluster randomised controlled trial | USA |
| Colon-Emeric, C. S.; McConnell, E.; Pinheiro, S. O.; et al | 2013 | CONNECT for better fall prevention in nursing homes: results from a pilot intervention study | Cluster randomised controlled trial | USA |
| Dilley, L. B.; Gray, S. M.; Zecevic, A.; et al | 2014 | An educational video to promote multi-factorial approaches for fall and injury prevention in long-term care facilities | Educational video evaluation | Canada |
| Eckstrom, E.; Neal, M. B.; Cotrell, V.; et al | 2016 | An Interprofessional Approach to Reducing the Risk of Falls Through Enhanced Collaborative Practice | Interprofessional education implementation - implementation science program | USA |
| Godlock, G. | 2016 | Implementation of an Evidence-Based Patient Safety Team to Prevent Falls in Inpatient Medical Units | Quality/ process improvement project/ EBP project following FOCUS-PDCA methodology | USA |
| Gray-Miceli, D.; Mazzia, L.; Crane, G. | 2016 | Advanced Practice Nurse-Led Statewide Collaborative to Reduce Falls in Hospitals | Randomised trial: Education collaborative - Quality improvement project | USA |
| Gygax Spicer, J. | 2017 | The Got-A-Minute Campaign to Reduce Patient Falls with Injury in an Acute Care Setting | Quality improvement | USA |
| Haralambous, B.; Haines, T. P.; Hill, K.; et al | 2010 | A protocol for an individualised, facilitated and sustainable approach to implementing current evidence in preventing falls in residential aged care facilities | Prospective cohort study protocol. Pre/post design in all RACFs. | Australia |
| Heck, J.; Gebhart, A.; Gaehle, K. E. | 2014 | Accountability and teamwork: tools for a fall-free zone | Quality improvement project report | USA |
| Hill, A. M.; McPhail, S. M.; Waldron, N.; et al | 2015 | Fall rates in hospital rehabilitation units after individualised patient and staff education programmes: a pragmatic, stepped-wedge, cluster-randomised controlled trial | Cluster-randomised, stepped-wedge controlled trial | Australia |
| Ireland, S.; Lazar, T.; Mavrak, C.; et al | 2010 | Designing a falls prevention strategy that works | Implementation of an EBP falls prevention strategy | Canada |
| Johnson, M.; Kelly, L; Siric, K.; et al | 2015 | Improving falls risk screening and prevention using an e-learning approach | Pre-post test design within two hospitals | Australia |
| Karnes, Michele J. | 2011 | Improving fall risk factor identification and documentation of risk reduction strategies by rehabilitation therapists through continuing education | Static group comparison design: pre-post cohort study (thesis) | USA |
| Kempegowda, P.; Chandan, J. S.; Hutton, R.; et al | 2018 | Focused educational intervention improves but may not sustain knowledge regarding falls management | Prospective study: pre-post cohort study | UK |
| Kent, F.; Courtney, J; Thorpe, J | 2018 | Interprofessional education workshops in the workplace for pre-registration learners: Aligning to National Standards | Quasi-experimental study to compare knowledge outcomes. | Australia |
| Lasater, K.; Cotrell, V.; McKenzie, G.; et al | 2016 | Collaborative Falls Prevention: Interprofessional Team Formation, Implementation, and Evaluation | Educational intervention: Quality improvement | USA |
| Leverenz, M.D.; Lape, J. | 2018 | Education on Fall Prevention to Improve Self-Efficacy of Nursing Staff in Long Term Care: A Pilot Study | Pre-test/ post test pilot study | USA |
| Lopez-Jeng, C.; Eberth, S. D. | 2019 | Improving Hospital Safety Culture for Falls Prevention Through Interdisciplinary Health Education | Pre-post cohort study | USA |
| Lugo, Irene | 2014 | Educating staff nurses on fall prevention strategies in hospice austin patients: Knowledge enhancement | Pre-post cohort study (Doctor of Nursing Practice thesis) | USA |
| Maloney, S.; Haas, R.; Keating, J. L.; et al | 2011 | Effectiveness of Web-based versus face-to-face delivery of education in prescription of falls-prevention exercise to health professionals: randomized trial | Head to head randomised trial design | Australia |
| McCarty, C.A.; Woehrle, T. A.; Waring, S.C.; et al | 2018 | Implementation of the MEDFRAT to Promote Quality Care and Decrease Falls in Community Hospital Emergency Rooms | Evidence-Based Quality Improvement project | USA |
| McConnell, E. S.; Lekan, D.; Bunn, M.; et al | 2009 | Teaching evidence-based nursing practice in geriatric care settings: the geriatric nursing innovations through education institute | Evidence based implementation project | USA |
| McKenzie, G.; Lasater, K.; Delander, G. E.; et al | 2017 | Falls prevention education: Interprofessional training to enhance collaborative practice | Quality improvement project | USA |
| Melin, C.M. | 2018 | Reducing falls in the inpatient hospital setting | Quality improvement project | USA |
| Meyer, G.; Kopke, S.; Haastert, B.; et al | 2009 | Comparison of a fall risk assessment tool with nurses' judgement alone: a cluster-randomised controlled trial | 12 month cluster-randomised controlled trial | Germany |
| Singh, I.; Okeke, J. | 2016 | Reducing inpatient falls in a 100% single room elderly care environment: evaluation of the impact of a systematic nurse training programme on falls risk assessment (FRA) | Quality improvement project | UK |
| Spiva, L.; Robertson, B.; Delk, M. Let al | 2014 | Effectiveness of team training on fall prevention | Longitudinal, repeated-measures design with intervention and comparison groups | USA |
| Szymaniak, S. | 2015 | Accurate falls risk assessment and interventions for preventing falls in patients in the acute care setting within a private hospital in a large capital city: a best practice implementation project | Quality improvement project | Australia |
| Teresi, J.A.; Ramirez, M.; Remler, D.; et al | 2013 | Comparative effectiveness of implementing evidence-based education and best practices in nursing homes: Effects on falls, quality-of-life and societal costs | Quasi-experimental cluster randomised trial | USA |
| Toye, C.; Kitchen, S.; Hill, A.; et al | 2017 | Piloting staff education in Australia to reduce falls in older hospital patients experiencing delirium | Quasi experimental, pre-test/ post test pilot cohort study. | Australia |
| Wheeler, E.; Coogle, C. L.; Fix, R. C.; et al | 2018 | Physical and Occupational Therapy Practice Improvement Following Interprofessional Evidence-Based Falls Prevention Training | Pre-post cohort study | USA |
| Williams, B.; Young, S.; Williams, D.; et al | 2011 | Effectiveness of a fall awareness and education program in acute care | Quasi experimental, pre-test/ post test study | Canada |
