# Supplementary material for: Education interventions for health professionals on falls prevention in health care settings: a 10-year scoping review
Source: BMC Geriatr. 2020 Nov 9;20:460. doi: 10.1186/s12877-020-01819-x (PMC7653707; doi:10.1186/s12877-020-01819-x)
Supplement: Supplementary file 4 — Additional file 4. Presage and planning elements of education interventions [file 12877_2020_1819_MOESM4_ESM.docx]

**Additional file 4: Presage and planning elements of education interventions**

| **Authors** | **Study location** | | | | **Learning environment** | | | | | | | **How were the teachers identified/ recruited?** | | | **Teachers qualified/ experienced** | **Teachers experienced in falls prevention** | **Evaluation planned from inception?** |
| --- | --- | --- | --- | --- | --- | --- | --- | --- | --- | --- | --- | --- | --- | --- | --- | --- | --- |
|  | Hospital only | LTC/ RACF | Hospital & LTC/ RACF | Other* | Ward | Classroom | Simulation centre | Online e-learning | Blended learning** | Other*** | LCF | Research team members | Independent educators | Recruited from/ as participants in study |  |  |  |
| Atkinson (2014) |  |  |  | 🗸 |  |  |  |  |  | 🗸 ^3^ |  | 🗸 |  |  | 🗸 | 🗸 | 🗸 |
| Becker (2011) |  | 🗸 |  |  |  |  |  |  |  |  | 🗸 |  |  | 🗸 | - | - | - |
| Brennan (2018) | 🗸 |  |  |  |  |  |  |  | 🗸 |  |  | 🗸 |  |  | - | 🗸 | 🗸 |
| Bursiek (2017) | 🗸 |  |  |  |  |  | 🗸 |  |  |  |  |  | 🗸 |  | 🗸 | - | 🗸 |
| Cabilan (2014) | 🗸 |  |  |  |  |  |  |  |  | 🗸 ^2^ |  |  |  |  | - | - | - |
| Campbell (2016) | 🗸 |  |  |  | 🗸 |  |  |  |  |  |  | 🗸 |  |  | - | - | 🗸 |
| Caton (2011) | 🗸 |  |  |  |  |  |  |  |  | 🗸 ^1^ |  |  |  | 🗸 | 🗸 | 🗸 | 🗸 |
| Colon-Emeric (2017) |  | 🗸 |  |  |  |  |  |  | 🗸 |  |  | 🗸 |  |  | 🗸 | - | 🗸 |
| Colon-Emeric (2013) |  | 🗸 |  |  |  |  |  |  | 🗸 |  |  | 🗸 |  |  | 🗸 | - | 🗸 |
| Dilley (2014) |  | 🗸 |  | 🗸 ^1^ |  |  |  |  |  | 🗸 ^1^ |  | 🗸 |  |  | 🗸 | 🗸 | - |
| Eckstrom (2016) |  |  | 🗸 |  |  |  |  |  |  | 🗸 |  | 🗸 |  |  | 🗸 | 🗸 | 🗸 |
| Godlock (2016) | 🗸 |  |  |  |  |  | 🗸 |  |  |  |  |  |  | 🗸 | - | 🗸 | 🗸 |
| Gray-Miceli (2016) | 🗸 |  |  |  |  |  |  |  |  | 🗸 |  | 🗸 |  |  | - | 🗸 | 🗸 |
| Gygax Spicer (2017) | 🗸 |  |  |  | 🗸 |  |  |  |  |  |  |  |  | 🗸 | 🗸 | 🗸 | - |
| Haralambous (2010) |  | 🗸 |  |  |  |  |  |  |  | 🗸 |  |  |  | 🗸 | - | - | 🗸 |
| Heck (2014) | 🗸 |  |  |  |  |  |  |  | 🗸 |  |  |  |  |  | - | - | - |
| Hill (2015) | 🗸 |  |  |  |  |  |  |  |  | 🗸 ^1^ |  | 🗸 |  |  | 🗸 | 🗸 | - |
| Ireland (2010) | 🗸 |  |  |  |  |  |  |  | 🗸 |  |  | 🗸 |  |  | - | - | 🗸 |
| Johnson (2015) | 🗸 |  |  |  |  |  |  | 🗸 |  |  |  |  | 🗸 |  | 🗸 | - | 🗸 |
| Karnes (2011) |  |  |  | 🗸 |  |  |  |  |  | 🗸 |  | 🗸 |  |  | - | - | 🗸 |
| Kempegowda (2018) | 🗸 |  |  |  |  |  |  |  |  | 🗸 |  | 🗸 |  |  | 🗸 | 🗸 | 🗸 |
| Kent (2018) |  |  |  | 🗸 |  |  |  |  |  | 🗸 |  |  |  |  | 🗸 | - | 🗸 |
| Lasater (2016) |  |  |  | 🗸 |  | 🗸 |  |  |  |  |  | 🗸 |  |  | 🗸 | 🗸 | 🗸 |
| Leverenz (2018) |  | 🗸 |  |  |  |  |  |  |  | 🗸 ^1^ |  | 🗸 |  |  | - | 🗸 | 🗸 |
| Lopez-Jeng (2019) | 🗸 |  |  |  |  |  |  |  |  | 🗸 ^1^ |  |  |  | 🗸 | - | 🗸 | 🗸 |
| Lugo (2014) | 🗸 |  |  |  |  |  |  |  |  | 🗸 ^1^ |  |  | 🗸 |  | - | - | - |
| Maloney (2011) |  |  | 🗸 | 🗸 ^1^ |  |  |  |  | 🗸 |  |  |  |  |  | 🗸 | 🗸 | 🗸 |
| McCarty (2018) | 🗸 |  |  |  |  |  |  |  |  | 🗸 ^1^ |  | 🗸 |  |  | - | - | 🗸 |
| McConnell (2009) | 🗸 |  |  |  |  |  |  |  | 🗸 |  |  | 🗸 |  |  | 🗸 | - | 🗸 |
| McKenzie (2017) |  |  | 🗸 |  |  | 🗸 |  |  |  |  |  |  |  |  | - | 🗸 | 🗸 |
| Melin (2018) | 🗸 |  |  |  |  |  |  |  | 🗸 ^1^ |  |  | 🗸 |  |  | - | - | 🗸 |
| Meyer (2009) |  | 🗸 |  |  |  |  |  |  |  | 🗸 ^1^ |  | 🗸 |  |  | 🗸 | 🗸 | 🗸 |
| Singh (2016) | 🗸 |  |  |  |  |  |  |  |  | 🗸 ^1^ |  | 🗸 |  |  | - | 🗸 | 🗸 |
| Spiva (2014) | 🗸 |  |  |  |  |  |  |  |  | 🗸 ^1^ |  | 🗸 |  |  | 🗸 | 🗸 | 🗸 |
| Szymaniak (2015) | 🗸 |  |  |  |  |  |  |  | 🗸 |  |  | 🗸 |  |  | - | 🗸 | 🗸 |
| Teresi (2013) |  | 🗸 |  |  |  |  |  |  |  | 🗸 ^1^ |  |  |  | 🗸 | - | - | 🗸 |
| Toye (2017) | 🗸 |  |  |  |  |  |  |  |  | 🗸 ^1^ |  | 🗸 |  |  | 🗸 | 🗸 | 🗸 |
| Wheeler (2018) |  | 🗸 |  |  |  |  |  |  |  | 🗸 |  |  |  |  | - | - | 🗸 |
| Williams (2011) | 🗸 |  |  |  | 🗸 |  |  |  |  |  |  |  | 🗸 |  | 🗸 | 🗸 | 🗸 |
| **Totals** | 23 | 9 | 3 | 6 | 3 | 2 | 2 | 1 | 9 | 21 | 1 | 22 | 4 | 7 | 18 | 21 | 32 |

*^1^ Study location was the community

**^1^ Blended learning environment was online/ face to face

***^1^ other learning environment was in-house; ^2^ learning environment was in the ward/ independent; ^3^ learning environment was at a conference
