# Supplementary material for: Education interventions for health professionals on falls prevention in health care settings: a 10-year scoping review
Source: BMC Geriatr. 2020 Nov 9;20:460. doi: 10.1186/s12877-020-01819-x (PMC7653707; doi:10.1186/s12877-020-01819-x)
Supplement: Supplementary file 5 — Additional file 5. Process elements of education interventions [file 12877_2020_1819_MOESM5_ESM.docx]

**Additional file 5: Process elements of education interventions**

| **Authors** | **Teachers trained in how to deliver the education program?** | **Learning objectives stated?** | **Learning objectives written in behavioural terms?** | **Recognition of learners' prior knowledge?** | | **Recognition of learners' prior experience?** | **Teaching & learning methods stated?** | **Teaching & learning methods aligned to learning objectives?** | **How long did the education program take to complete?** |
| --- | --- | --- | --- | --- | --- | --- | --- | --- | --- |
|  |  |  |  | Formal | Informal |  |  |  |  |
| Atkinson (2014) | 🗸 | 🗸 | 🗸 | 🗸 |  | 🗸 | 🗸 | 🗸 | 90' workshop, initial 15 min lecture reviewing critical elements of gait and falls risk assessment. Participants rotated through each of the four stations for the next 60mins. |
| Becker (2011) | - | - | - | - |  | - | - | - | 1 day |
| Brennan (2018) | - | 🗸 | 🗸 |  | 🗸 | - | 🗸 | 🗸 | 20mins |
| Bursiek (2017) | - | 🗸 | 🗸 | - | - | - | 🗸 | 🗸 | Scenario 1: 15mins scenario, 30mins debrief; Scenario 2: 15mins scenario, 45mins debrief |
| Cabilan (2014) | - | - | - | - | - | - | 🗸 | 🗸 | 15-20mins |
| Campbell (2016) | - | 🗸 | 🗸 | 🗸 |  | 🗸 | 🗸 | - | Not stated |
| Caton (2011) | 🗸 | 🗸 | 🗸 | 🗸 |  | 🗸 | 🗸 | 🗸 | 1 hour lecture, duration of other teaching and learning activities not stated |
| Colon-Emeric (2017) | 🗸 | 🗸 | - | - | - | 🗸 | 🗸 | 🗸 | Numerous sessions of varying length ranging from 20 minutes to 4 hours |
| Colon-Emeric (2013) | 🗸 | 🗸 | - | - | - | 🗸 | 🗸 | 🗸 | Half-day training session followed by 11 weekly teleconferences |
| Dilley (2014) | - | 🗸 | - |  | 🗸 | 🗸 | 🗸 | 🗸 | 1 x 7', 1 x 12' video |
| Eckstrom (2016) | 🗸 | 🗸 | 🗸 | 🗸 |  | 🗸 | 🗸 | 🗸 | 4-hour training workshop plus “coaching” for implementation for one year |
| Godlock (2016) | 🗸 | - | - | 🗸 |  | 🗸 | 🗸 | 🗸 | Classes offered over 5 days during day and night shifts. 30' session. The perfect Storm Simulation Team STEPPS training was 3.5 hours long. |
| Gray-Miceli (2016) | 🗸 | - | - |  | 🗸 | 🗸 | 🗸 | 🗸 | 1.5 days (12 hours) delivered in 3 complete cycles |
| Gygax Spicer (2017) | 🗸 | - | - |  | 🗸 | 🗸 | 🗸 | 🗸 | Not stated |
| Haralambous (2010) | - | 🗸 | 🗸 | - | - | - | - | 🗸 | FRNs: one day training program on falls prevention, action research, practice change and other aspects of the project, such as data collection processes. |
| Heck (2014) | - | - | - |  | 🗸 | 🗸 | 🗸 | 🗸 | Not stated |
| Hill (2015) | 🗸 | - | - | - | - | - | 🗸 | 🗸 | 6 h of online video conference-based training. Face to face staff training in the week of the start of the intervention. |
| Ireland (2010) | - | - | - | - | - | 🗸 | 🗸 | 🗸 | Not stated |
| Johnson (2015) | - | - | - | 🗸 |  | 🗸 | 🗸 | 🗸 | 60mins |
| Karnes (2011) | 🗸 | 🗸 | 🗸 |  | 🗸 | 🗸 | 🗸 | 🗸 | 2.5 hour session |
| Kempegowda (2018) | - | 🗸 | - | - | - | 🗸 | 🗸 | 🗸 | 5 mins delivered weekly |
| Kent (2018) | 🗸 | 🗸 | - | - | - | 🗸 | 🗸 | 🗸 | 2 hour workshop |
| Lasater (2016) | 🗸 | - | - | - | - | - | 🗸 | 🗸 | 4 hours |
| Leverenz (2018) | 🗸 | 🗸 | - | 🗸 |  | 🗸 | 🗸 | 🗸 | 6 x 30 mins presentations plus 30min staff interactions |
| Lopez-Jeng (2019) | 🗸 | 🗸 | - | 🗸 |  | 🗸 | 🗸 | 🗸 | 4 x 1 hour |
| Lugo (2014) | - | 🗸 | 🗸 | 🗸 |  | 🗸 | 🗸 | 🗸 | Not stated -offered at the beginning of monthly staff meeting, 30-min breaks were built into the educational agenda, and question time at end |
| Maloney (2011) | 🗸 | 🗸 | - | - | - | - | 🗸 | 🗸 | 1 day seminar or web-based equivalent educational material over a 4-week period. |
| McCarty (2018) | - | - | - | - | - | - | 🗸 | 🗸 | 1 hour education session |
| McConnell (2009) | - | 🗸 | 🗸 | 🗸 |  | 🗸 | 🗸 | 🗸 | 6-9 months, 39 contact hour. |
| McKenzie (2017) | 🗸 | 🗸 | 🗸 |  |  | 🗸 | 🗸 | 🗸 | 4 hours -2 hours of large group training on evidence based approaches to falls prevention, 2 hour individualised team planning and coaching session. |
| Melin (2018) | - | 🗸 | 🗸 | - | - | - | 🗸 | 🗸 | 20min Power Point presentation |
| Meyer (2009) | 🗸 | - | - | - | - | - | 🗸 | 🗸 | 60-90mins |
| Singh (2016) | - | 🗸 | - | - | - | 🗸 | 🗸 | 🗸 | Delivered fortnightly, each session 45mins to ensure that each staff member attended at least one teaching session over 12 months. |
| Spiva (2014) | 🗸 | 🗸 | - | - | - | - | 🗸 | 🗸 | 3 x 30min training sessions |
| Szymaniak (2015) | 🗸 | 🗸 | - |  | 🗸 | 🗸 | 🗸 | 🗸 | Online training 17-20mins, rest not stated. |
| Teresi (2013) | 🗸 | 🗸 | 🗸 | 🗸 |  | 🗸 | 🗸 | 🗸 | Unsure |
| Toye (2017) | 🗸 | 🗸 | - | - | - | - | 🗸 | 🗸 | 30mins |
| Wheeler (2018) | - | 🗸 | 🗸 |  | 🗸 | - | - | 🗸 | 6 x 2 hour face-to-face training sessions covering 7 learning modules. |
| Williams (2011) | - | - | - | - | - | 🗸 | 🗸 | - | Monthly falls meeting approx 20mins in length that included discussion-teaching session on key concept. |
