# Supplementary material for: Education interventions for health professionals on falls prevention in health care settings: a 10-year scoping review
Source: BMC Geriatr. 2020 Nov 9;20:460. doi: 10.1186/s12877-020-01819-x (PMC7653707; doi:10.1186/s12877-020-01819-x)
Supplement: Supplementary file 6 — Additional file 6. Teaching and learning activities [file 12877_2020_1819_MOESM6_ESM.docx]

**Additional file 6: Teaching and learning activities**

| **Teaching/ learning category** | **Sub categories** | **No. of papers** | **Paper ID** |
| --- | --- | --- | --- |
| **Methods of delivery** | Didactic lectures/ formal delivery | 22 | [1-22] |
|  | Other oral presentation e.g. in-service training | 8 | [1, 8, 23-28] |
|  | E-learning/ online | 8 | [6, 8, 14, 24, 27, 29-31] |
|  | Self-directed learning | 8 | [1, 14, 29-33] |
|  | Video presentation/ demonstration | 8 | [1, 12, 15, 20, 26, 29, 33, 34] |
| **Interactive learning activities/ experiential learning** | Group -based learning activities (e.g. team presentations, problem solving, brainstorming) | 19 | [1, 7-9, 11, 14, 15, 17, 18, 21, 24-26, 28, 34-38] |
|  | Debriefing sessions/ reflective dialogue | 17 | [7, 8, 11, 14, 17-19, 24, 26, 28, 30, 32, 35-39] |
|  | Station-based activities | 1 | [1] |
|  | Case studies/ clinical scenarios (paper-based) | 9 | [1, 3, 6, 12, 17, 18, 21, 32, 37] |
|  | Case studies/ clinical scenarios (video) | 5 | [1, 9, 12, 19, 33] |
|  | Role play | 2 | [14, 32] |
|  | Simulation | 2 | [35, 39] |
|  | Skills training | 15 | [1, 7-9, 11, 14, 18, 20, 21, 24, 26, 28, 30, 35, 38] |
|  | Interactive learning activities (details not described) | 8 | [6, 7, 11, 23, 24, 28, 31, 38] |
| **Supported learning** | Individual mentoring/ coaching or personal feedback | 9 | [1, 5, 6, 8, 14, 15, 25, 30, 38] |
|  | Bedside coaching | 1 | [2] |
|  | Peer to peer discussion and feedback/ staff huddles | 11 | [8, 13, 15, 17, 21, 24, 25, 28, 35-37] |
|  | Staff meetings | 2 | [24, 28] |
|  | Team coaching | 4 | [8, 15, 25, 26] |
| **Written learning material** | Handouts | 4 | [12, 18, 27, 34] |
|  | Resource folders | 3 | [2, 3, 38] |
|  | Falls assessment tool | 6 | [3, 13, 18, 27, 31, 35] |
|  | Poster | 1 | [28] |
| **Other** | Teleconferences | 1 | [6] |
| **Assessments** | Practical assessment | 1 | [5] |
|  | Knowledge assessment | 6 | [3, 23, 29-31, 37] |
