# Supplementary material for: Education interventions for health professionals on falls prevention in health care settings: a 10-year scoping review
Source: BMC Geriatr. 2020 Nov 9;20:460. doi: 10.1186/s12877-020-01819-x (PMC7653707; doi:10.1186/s12877-020-01819-x)
Supplement: Supplementary file 7 — Additional file 7. Product elements of education interventions [file 12877_2020_1819_MOESM7_ESM.docx]

**Additional file 7: Product elements of education interventions**

| **Authors** | **Clinical outcomes** | **Educational outcomes** | **Assessment of learners’ achievement of learning objectives** | **Evaluation of education program conducted?** | **How was the evaluation data gathered?** | **Kirkpatrick level/s of evaluation reported?** | | | | |  |
| --- | --- | --- | --- | --- | --- | --- | --- | --- | --- | --- | --- |
|  |  |  |  |  |  | **Level 1** | **Level 2** | **Level 3** | **Level 4** | **Not stated** |  |
| Atkinson (2014) | - | 🗸 | 🗸 | 🗸 | Survey data on perceived competence | 🗸 | 🗸 |  |  |  |  |
| Becker (2011) | 🗸 | - | - | - | - |  |  |  |  |  |  |
| Brennan (2018) | 🗸 | 🗸 | 🗸 | 🗸 | Post fall prevention education questionnaire |  |  | 🗸 |  |  |  |
| Bursiek (2017) | 🗸 | 🗸 | 🗸 | 🗸 | Self reports scales: Professional Practice Environment Assessment Scale and Mayo High Performance Teamwork Scale. Measured before training, 2mths and 6mths post education. No education evaluation. |  |  | 🗸 |  |  |  |
| Cabilan (2014) | 🗸 | - | 🗸 | - | - |  |  | 🗸 |  |  |  |
| Campbell (2016) | - | 🗸 | 🗸 | 🗸 | Pre/ post knowledge test; Ongoing process evaluation |  | 🗸 |  |  |  |  |
| Caton (2011) | 🗸 | 🗸 | 🗸 | 🗸 | pre-post test, observation, process monitoring, EMR data, manual audit | 🗸 | 🗸 | 🗸 |  |  |  |
| Colon-Emeric (2017) | 🗸 | 🗸 | 🗸 | 🗸 | Previously validated scales: communication openness, accuracy, and timeliness; Participation in Decision-Making Instrument; Safety Organizing Scale; Local Interaction Scale, Perceived Quality of Care Scale. |  |  | 🗸 | 🗸 |  |  |
| Colon-Emeric (2013) | 🗸 | 🗸 | 🗸 | 🗸 | Previously validated scales: communication openness, accuracy, and timeliness; Participation in Decision-Making Instrument; Safety Organizing Scale; Local Interaction Scale and Perceived Quality of Care Scale. Facility fall rates as an exploratory measure. |  |  | 🗸 |  |  |  |
| Dilley (2014) | - | - | - | - | Not stated |  |  |  |  |  |  |
| Eckstrom (2016) | 🗸 | 🗸 | 🗸 | 🗸 | Observation of interventions; pre-post test of K/ A/ S; Evaluation using: STEADI questionnaire; Stay Independent questionnaire; | 🗸 | 🗸 | 🗸 |  |  |  |
| Godlock (2016) | 🗸 | 🗸 | 🗸 | 🗸 | Feedback from training baseline for future Team STEPPS training and used to focus staff education on most common deficiencies seen in fall simulation. Surveys pre and post simulation. |  | 🗸 |  | 🗸 |  |  |
| Gray-Miceli (2016) | 🗸 | - | - | - | Fall rates per 1000 bed days; facility and unit-level variables. |  |  | 🗸 |  |  |  |
| Gygax Spicer (2017) | 🗸 | - | - | - | Fall rates per 1000 bed days. |  |  |  | 🗸 |  |  |
| Haralambous (2010) | 🗸 | - | 🗸 | - | Fall rates per 1000 bed days. |  |  |  | 🗸 |  |  |
| Heck (2014) | 🗸 | - | - | - | Incidence of falls |  |  |  | 🗸 |  |  |
| Hill (2015) | 🗸 | - | - | - | Only process outcomes measured on the number of education sessions delivered |  |  |  | 🗸 |  |  |
| Ireland (2010) | 🗸 | 🗸 | 🗸 | 🗸 | Completion data from e-learning platform |  | 🗸 |  |  |  |  |
| Johnson (2015) | 🗸 | 🗸 | 🗸 | 🗸 | Documentation of falls risk assessment, falls prevention strategies and post fall management; pre-post survey, falls and injury rates |  | 🗸 | 🗸 | 🗸 |  |  |
| Karnes (2011) | 🗸 | 🗸 | 🗸 | 🗸 | Chart audits |  |  | 🗸 |  |  |  |
| Kempegowda (2018) | 🗸 | 🗸 | 🗸 | 🗸 | Pre and post knowledge surveys |  | 🗸 | 🗸 |  |  |  |
| Kent (2018) | 🗸 | 🗸 | 🗸 | 🗸 | Knowledge tests and evaluation forms. | 🗸 | 🗸 |  |  |  |  |
| Lasater (2016) | 🗸 | 🗸 | 🗸 | 🗸 | Feedback from a. coaching plans devised by practice teams with assistance from team members, b. interim team reports, c. post intervention practice team member interviews. Feedback from staff interviews a. appreciation of IP approach, b. strategies for engaging staff, c. need for leadership presence. |  |  |  |  |  |  |
| Leverenz (2018) | 🗸 | 🗸 | 🗸 | 🗸 | Self-efficacy for preventing falls-assistant scale (SEPF-A); 1 item self-efficacy for preventing falls-nurse (SEPF-N) scale. Post-training survey, to obtain demographic information from the nursing staff, assess satisfaction with the training, measure the collaborative nature of the training, and gather additional self-efficacy data. Qualitative feedback of individual and group training sessions. | 🗸 | 🗸 | 🗸 |  |  |  |
| Lopez-Jeng (2019) | 🗸 | 🗸 | 🗸 | 🗸 | Post-test surveys used to measure participant outcomes and AHRQ Hospital Survey on Patient Safety Culture measured safety culture in the hospital pre and post intervention. | 🗸 | 🗸 | 🗸 |  |  |  |
| Lugo (2014) | - | 🗸 | 🗸 | 🗸 | Pre and post knowledge surveys. |  | 🗸 |  |  |  |  |
| Maloney (2011) | 🗸 | 🗸 | 🗸 | 🗸 | Various -knowledge tests, assignments self-reported change in practice, self-reported satisfaction, and ratings of relevance of program to current work roles, time spent engaged with the learning resources. | 🗸 | 🗸 | 🗸 |  |  |  |
| McCarty (2018) | - | 🗸 | - | 🗸 | Satisfaction with training | 🗸 |  |  |  |  |  |
| McConnell (2009) | - | 🗸 | 🗸 | 🗸 | Questionnaires -pre-post testing, post-education self-efficacy and self-reported implementation in practice | 🗸 | 🗸 |  |  |  |  |
| McKenzie (2017) | - | - | 🗸 | 🗸 | Knowledge questionnaire and post workshop survey |  |  |  |  |  |  |
| Melin (2018) | 🗸 | - | - |  | Falls rates |  |  |  | 🗸 |  |  |
| Meyer (2009) | 🗸 | - | - |  | Fall incidence data |  |  | 🗸 | 🗸 |  |  |
| Singh (2016) | 🗸 | - | 🗸 | 🗸 | Audit on compliance of the FRAT; fall incidence per 1000 bed days |  |  | 🗸 | 🗸 |  |  |
| Spiva (2014) | 🗸 | 🗸 | 🗸 | 🗸 | Numerous study questionnaires -teamwork and communication measures, practice audits, falls and fall related injury rates |  | 🗸 | 🗸 | 🗸 |  | |
| Szymaniak (2015) | 🗸 | 🗸 | 🗸 | 🗸 | Practice and documentation audit, field notes, falls rates and injury rates |  | 🗸 | 🗸 | 🗸 |  | |
| Teresi (2013) | 🗸 | 🗸 | 🗸 | 🗸 | Pre-post knowledge tests before and after each module, number of staff trained, implementation audits -number of falls and other Nursing Home resident outcomes. |  | 🗸 | 🗸 | 🗸 |  | |
| Toye (2017) | 🗸 | 🗸 | 🗸 | 🗸 | Questionnaires -knowledge change and practice change, focus groups, implementation audits, falls and injury rates | 🗸 | 🗸 | 🗸 | 🗸 |  | |
| Wheeler (2018) | 🗸 | 🗸 | 🗸 | 🗸 | Practice change outcomes -consensus review process for abstracting information contained in medical charts. |  |  |  |  |  | |
| Williams (2011) | 🗸 | 🗸 | 🗸 | 🗸 | pre and post-test survey |  | 🗸 |  |  |  | |
| Totals | 32 | 27 | 30 | 29 |  | 10 | 19 | 20 | 14 | 0 | |
